# Preoperative patient risk factors for intraoperative hypotension: a systematic review and meta-analysis

**DOI:** 10.3389/fcvm.2025.1709004

**Published:** 2025-12-05

**Authors:** Nils Daum, Daniel Bill, Moritz Thiele, Julian Felber, Dario von Wedel, Claudia Spies, Felix Balzer, Rudolf Mörgeli, Oliver Hunsicker, Anika Müller, Dennis Contag, Anne Pohrt, Annika Bald, Max Kayser, Sascha Treskatsch, Maximilian Markus

**Affiliations:** 1 Department of Anesthesiology and Intensive Care Medicine (CCM/CVK), Charité – Universitätsmedizin Berlin, Corporate Member of Freie Universität Berlin and Humboldt, Universität zu Berlin, Berlin, Germany; 2 Institute of Medical Informatics, Charité—Universitätsmedizin Berlin, Corporate Member of Freie Universität Berlin and Humboldt Universität zu Berlin, Berlin, Germany; 3 Institute for Biometry and Clinical Epidemiology, Charité—Universitätsmedizin Berlin, Corporate Member of Freie Universität Berlin and Humboldt Universität zu Berlin, Berlin, Germany; 4 Department of Anesthesiology and Intensive Care Medicine (CBF), Charité – Universitätsmedizin Berlin, Corporate Member of Freie Universität Berlin and Humboldt, Universität zu Berlin, Berlin, Germany

**Keywords:** intraoperative hypotension, preoperative risk factors, patient characteristics, perioperative management, cardiovascular risk, meta-analysis

## Abstract

**Background:**

Intraoperative hypotension (IOH) presents a risk factor for postoperative organ dysfunction. However, as a unique definition of IOH is still missing, the influence of individual preoperative patient characteristics on IOH remains poorly understood. This systematic review aimed to examine the variability in IOH definitions and to identify preoperative risk factors associated with IOH.

**Methods:**

A systematic literature search was conducted from inception to March 2, 2024. Studies reporting on IOH and from which the association between preoperative characteristics and IOH in cardiac and non-cardiac surgery could be derived were included. Odds ratios (ORs) were either extracted directly or calculated based on available patient-level data. Pooled estimates were generated using a random-effects model.

**Results:**

Out of 7,361 screened studies, 78 met the inclusion criteria. Heterogeneity was high due to varying IOH definitions. 14 preoperative factors were included in the meta-analysis. Older age (OR 1.03, 95% CI 1.02–1.04) and female sex (OR 1.16, 95% CI 1.08–1.24) were associated with increased IOH risk. ASA-II was linked to lower risk compared to ASA-III (OR 0.80, 95% CI 0.70–0.91). Diabetes mellitus (OR 1.18, 95% CI 1.04–1.35) and arterial hypertension (OR 1.56, 95% CI 1.33–1.83) were independent predictors. ACE inhibitor use (angiotensin-converting enzyme inhibitor use; OR 1.63, 95% CI 1.42–1.88), angiotensin receptor blocker (ARB) use (OR 1.38, 95% CI 1.01–1.89), and emergent surgery (OR 1.25, 95% CI 1.09–1.42) also increased IOH incidence. The risk of bias was low to moderate.

**Conclusion:**

The substantial variability in IOH definitions and several preoperative IOH influencing patient characteristics highlight the need for standardized criteria to improve comparability and guide personalized perioperative management.

**Systematic Review Registration:**

identifier PROSPERO CRD42024514229.

## Introduction

Intraoperative hypotension (IOH) presents a risk factor for serious postoperative complications, including major adverse cardiovascular events (MACE), stroke, acute kidney injury (AKI), and increased perioperative mortality (1, 2). Evidence indicates that postoperative organ dysfunction is not only associated with the occurrence of hypotension, but is even more strongly linked to its severity and duration, with both brief but profound hypotension as well as prolonged mild hypotension significantly contributing to risk (3).

As IOH is not yet uniformly defined, varying thresholds and durations applied across studies result in inconsistent incidence rates and limited comparability of findings (4). A comprehensive review highlighted the lack of a consensus definition and suggested a classification based on underlying mechanisms such as vasodilation, hypovolemia, or myocardial depression, emphasizing the need for individualized clinical interpretation (5). Other works have stressed the complex and multifactorial pathophysiology of IOH, calling attention to both systemic and patient-specific contributors (6). More recent perspectives have advocated for a symptom-oriented understanding of IOH, shifting the focus from fixed blood pressure thresholds to the broader hemodynamic impact, potentially redefining intraoperative blood pressure management (7).

In this context, emerging data suggest that specific patient-related factors may increase susceptibility to IOH. A retrospective cohort study identified preoperatively assessed functional status, measured using the Fried frailty criteria (8), as an independent predictor of IOH in elderly non-cardiac surgical patients (9). In addition, a systematic review demonstrated that preoperative volume status may also significantly influence the risk of IOH (10). A subgroup analysis pointed toward potentially relevant gender-related susceptibilities, suggesting that elderly female patients might be particularly vulnerable to IOH (11).

## Objective

This systematic review aimed to examine the variability in IOH definitions used across studies, as inconsistent definitions may affect the identification and comparability of preoperative risk factors. Furthermore, we aimed to identify preoperative patient-related characteristics that serve as risk factors for the incidence of IOH. We hypothesized that patient-specific factors can be identified before surgery that are consistently associated with an increased risk of IOH.

## Methods

### Protocol and registration

The protocol for this systematic review was registered in PROSPERO on 01.03.2024 (CRD42024514229) (12). The study was conducted in accordance with the Preferred Reporting Items for Systematic Reviews and Meta-Analyses (PRISMA) guidelines (13).

### Selection criteria

Prior to the systematic search, inclusion criteria were established through a consensus-based approach using the PICOS framework (Participants, Interventions, Comparisons, Outcomes, and Study Design) (Table 1).

**Table 1:** PICOS criteria for the inclusion criteria of the systematic literature review.

**P**	Patients (≥18 years old) undergoing cardiac and non-cardiac surgery
**I**	Preoperative patient characteristics as predictors for hypotensive events (e.g., age, ASA status, pre-existing conditions, premedication)
**C**	Patients without the potential risk factors described above
**O**	Hypotensive event (e.g., Probability of Occurrence, Rate, Duration, and Severity)
**S**	Randomized controlled trials, Prospective cohort studies, Retrospective cohort studies

ASA, American society of anesthesiologists; PICOS, participants, interventions, comparisons, outcomes, study design.

Studies were excluded if they were not published in English or German language.

All authors collaboratively developed the systematic search strategy. Following its approval, the search was conducted in databases including Embase, MEDLINE (via Ovid) and the Cochrane Library. The detailed search strategy for each database is provided in Supplementary Table S1 in the Supplementary Appendix. The search covered all records available from the inception of each database up to March 2, 2024. Additionally, we manually screened the reference lists of relevant review articles to identify further eligible studies.

### Study selection

Two independent and blinded reviewers screened all studies retrieved from the systematic search, assessing titles and abstracts based on the PICOS criteria. A third independent reviewer resolved discrepancies. After title and abstract screening, the same procedure was applied to the full-text assessment.

### Data extraction process

The full texts of all included studies were reviewed by one researcher, who extracted all relevant information. A second researcher cross-checked the extracted data for accuracy and consistency. Extracted Information included the PICOS criteria as well as patient and study specific characteristics, and the definition of IOH used. Given the primary objective of this review to examine the variability in IOH definitions, we did not impose a uniform blood pressure threshold. Instead, IOH was extracted as reported in the original study, provided that the authors explicitly defined the event as intraoperative in nature. For meta-analytic pooling, IOH was therefore operationalized as a binary outcome (occurrence vs. no occurrence) based on the study's definition. This approach reflects the real-world heterogeneity of IOH definitions and enables comparison of patientś susceptibility rather than of specific hemodynamic thresholds.

### Risk of bias assessment

The risk of bias was assessed using the RoB-2 tool (14) for randomized controlled studies, ROBINS-E tool (15) for prospective studies and the Newcastle-Ottawa Scale (16) for retrospective studies. Two raters independently evaluated each study. In case of discrepancies, a third rater determined the final overall risk of bias.

### Statistical analysis

Statistical analysis was performed with MetaAnalysisOnline.com (17). The respective odds ratios (OR) for the incidence of IOH were pooled using a random-effects model, particularly for studies exhibiting high heterogeneity. Whenever available, ORs were extracted directly from the studies. In cases where ORs were missing, they were independently calculated using logistic regression models based on individual patient data, provided sufficient data was available.

Patient characteristics identified in individual studies as influencing the incidence of IOH were included in the meta-analysis, if data were available from at least five independent studies. This criterion was applied to mitigate potential bias and ensure the robustness of the analysis. Subgroup analyses were conducted for studies that exclusively investigated either general anesthesia or spinal anesthesia. Studies that included both anesthetic techniques or focused solely on regional anesthesia were categorized as “Other”. For the age-related meta-analysis, studies with age-restricted populations (e.g., cohorts limited to patients above a predefined age threshold) were excluded to prevent range restriction bias, unless sufficient within-study age variability was reported. To prevent demographic confounding, obstetric studies were excluded from the meta-analyses assessing sex and age, as these populations are uniformly female and younger. Obstetric studies were retained in other analyses, where demographic imbalance does not structurally bias effect estimates.

Inter-study heterogeneity was assessed using the *I*^2^ statistic (values > 75% indicating considerable heterogeneity, values <25% suggesting low heterogeneity) and *τ*^2^, estimated via the restricted maximum-likelihood method, and *p*-values derived from Cochran's *Q* test (18, 19). Potential publication bias was assessed using a funnel plot to visually examine left–right symmetry (20). A 95% confidence interval (95% CI) was applied, and statistical significance was defined as *p* < 0.05.

## Results

### Results of the literature search

**Figure 1:**
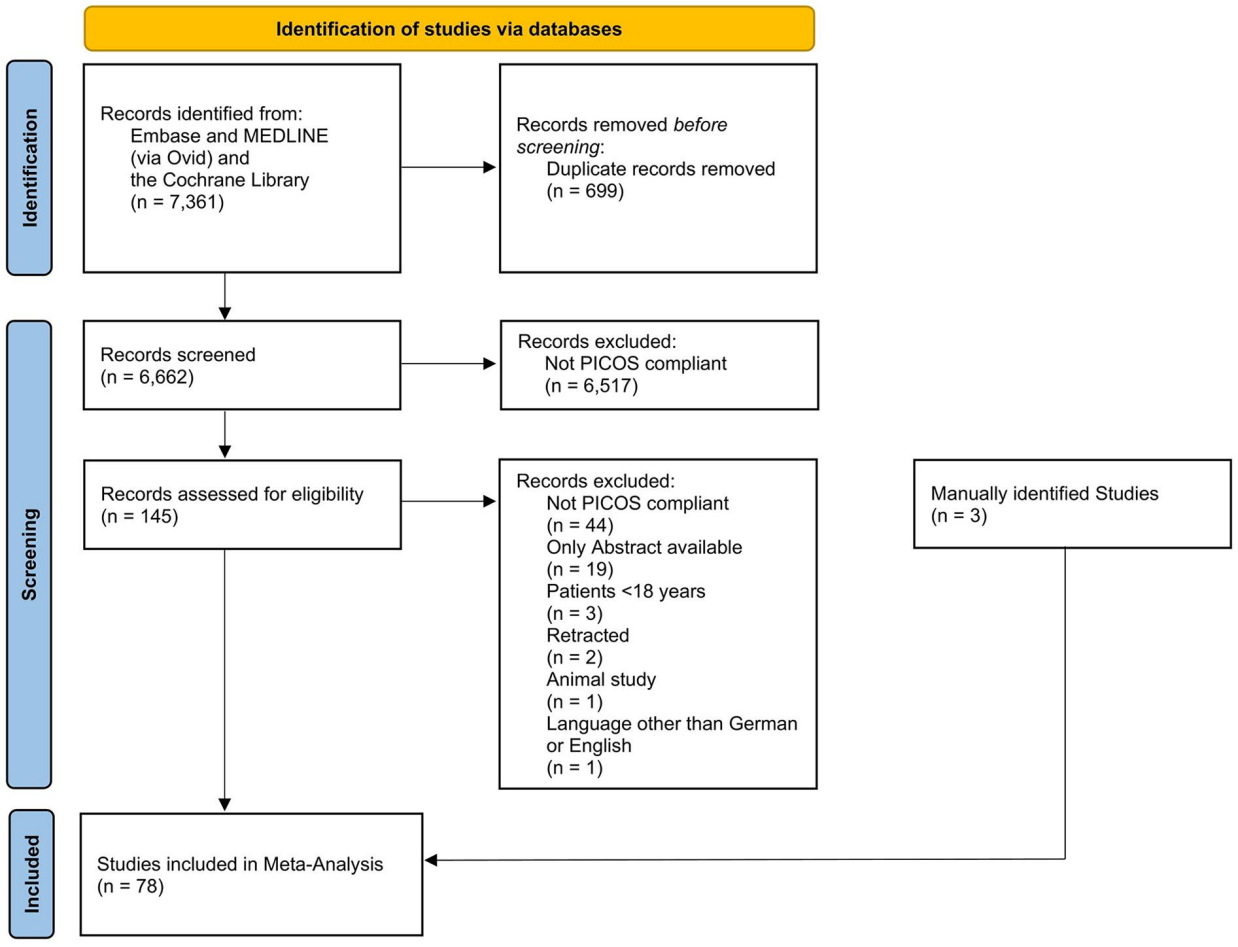
Flowchart of the systematic search.

Of the 7,361 screened studies, 78 met the inclusion criteria and were included in the statistical analysis (Figure 1). The included studies comprised a total of 934,021 patients, of whom one study followed a randomized controlled design, 46 a prospective study design, while 31 studies were retrospective (Table 2). A total of 45 studies investigated patients under general anesthesia, whereas six studies included both general and spinal anesthesia. An additional 21 studies exclusively focused on spinal anesthesia, while two studies examined a combination of general and regional anesthesia, and two studies solely assessed regional anesthesia. A total of 48 studies focused exclusively on elective surgeries, whereas 18 studies analyzed both elective and emergency procedures.

**Table 2:** Study characteristics along with the respective definitions of intraoperative hypotension.

Author	Year	Country	General/Spinal/Regional Anesthesia	Blood Pressure Definition of IOH	Patient Total	LoE	Women (%)	Emergency/Elective	Main surgery department
Abdelhamid et al. (a) (21)	2022	Egypt	Spinal	MAP <75%	71	3	28 (39.4%)	Elective	Orthopedic Surgery
Abdelhamid et al. (b) (22)	2022	Egypt	General	MAP <75%	93	3	44 (47.3%)	Elective	Orthopedic Surgery
Aissaoui et al. (23)	2022	Morocco	General	SAP <90 mmHg or SAP <70% or MAP <65 mmHg or MAP <70%	64	3	28 (43.8%)	Elective	General Surgery
Aktas Yildirim et al. (24)	2023	Turkey	General	SAP <90 mmHg or MAP <70%	85	3	22 (25.9%)	Elective	General Surgery
Alghanem et al. (25)	2020	Jordan	General/Spinal	SAP <70%	502	4	237 (47.2%)	n.a.	Orthopedic Surgery
Au et al. (26)	2016	USA	General	SAP <90 mmHg	40	3	21 (53.0%)	Elective	Orthopedic Surgery
Baek et al. (27)	2023	South Korea	Regional	SAP <90 mmHg or MAP <60 mmHg	2,152	4	1,003 (46.6%)	Elective	Orthopedic Surgery
Bellotti et al. (28)	2022	USA	General	MAP <55 mmHg	857	4	525 (61.3%)	Both	General Surgery
Bijker et al. (2)	2009	Netherlands	General/Spinal	SAP <80 mmHg	1,705	3	825 (48.4%)	Elective	General Surgery/Vascular Surgery
Bishop et al. (29)	2017	South Africa	Spinal	SAP <90 mmHg	504	3	504 (100%)	Both	Obstetrics
Boyle et al. (30)	2022	Canada	General	MAP <80 mmHg	47	3	14 (29.8%)	Elective	Neurosurgery
Brenck et al. (31)	2009	Germany	Spinal	SAP <90 mmHg or MAP <80%	503	4	503 (100%)	Both	Obstetrics
Casalino et al. (32)	2006	Italy	General	MAP ≤60 mmHg or MAP <70%	144	4	41 (28.5%)	Elective	Cardiac Surgery
Chen et al. (33)	2023	China	General	MAP <60 mmHg or MAP <80%	173	3	75 (43.4%)	Elective	General Surgery
Cheung et al. (34)	2015	Canada	General/Regional	SAP <90 mmHg or MAP <65%	193	3	128 (66.3%)	Elective	General Surgery
Chiang et al. (35)	2022	Taiwan	General	SAP <80 mmHg	1,833	4	781 (42.6%)	Elective	Neurosurgery
Chinachoti et al. (36)	2007	Thailand	Spinal	SAP <80%	2,000	3	1,338 (66.9%)	Both	Orthopedic Surgery
Choi et al. (37)	2020	South Korea	General	MAP <65 mmHg or MAP <70%	77	3	36 (46.8%)	Elective	General Surgery
Chowdhury et al. (38)	2022	India	Spinal	MAP <80%	50	3	12 (24.0%)	Elective	General Surgery
Chumpathong et al. (39)	2006	Thailand	Spinal	SAP ≤100 mmHg	991	4	991 (100%)	Both	Obstetrics
Czajka et al. (40)	2023	Poland	General	MAP ≤65 mmHg	508	3	269 (53.0%)	Both	General Surgery
Dai et al. (41)	2020	China	General/Spinal	SAP <90 mmHg or SAP <80%	5,864	4	3,103 (52.9%)	Both	n.a.
Doo et al. (42)	2021	South Korea	Regional	SAP <90 mmHg or SAP <70%	116	4	52 (44.8%)	Elective	Orthopedic Surgery
Elbadry et al. (43)	2022	Egypt	Spinal	MAP <65 mmHg or MAP <80%	55	3	55 (100%)	Elective	Obstetrics
Fathy et al. (44)	2023	Egypt	General	SAP <70% or MAP <65 mmHg	153	3	83 (54.2%)	Elective	Neurosurgery
Fukuhara et al. (45)	2021	Japan	General/Spinal	SAP ≤80 mmHg	245	4	43 (16.9%)	n.a.	Urology
Gregory et al. (46)	2021	USA	n.a.	MAP ≤65 mmHg	368,222	4	226,694 (62.0%)	n.a.	Orthopedic Surgery
Gurunathan et al. (47)	2024	Australia	General	SAP <70% or MAP <55 mmHg	537	3	207 (38.5%)	Elective	n.a.
Hartmann et al. (48)	2002	Germany	Spinal	MAP <70%	3,098	4	1,180 (38.1%)	Both	Orthopedic Surgery
Hojo et al. (49)	2022	Japan	General	MAP ≤55 mmHg	395	4	184 (46.6%)	n.a.	Oral and maxillofacial Surgery
Hoppe et al. (50)	2022	Germany	General	MAP <70%	366	3	195 (53.0%)	Elective	Ear, Nose and Throat Surgery
Huang et al. (51)	2024	China	General	SAP <70% or MAP < 80% or SAP <90 mmHg and MAP <60 mmHg	112	3	44 (38.3%)	Elective	n.a.
Jia et al. (52)	2022	China	General	Blood pressure <80%	367	4	48 (13.1%)	n.a.	Vascular Surgery
Jin et al. (53)	2021	China	General	MAP <65 mmHg	114	4	66 (57.9%)	n.a.	General Surgery
Jin et al. (54)	2024	China	General	SAP <70% or MAP <60 mmHg or MAP <70%	95	3	63 (66.3%)	Elective	General Surgery
Jor et al. (55)	2018	Czech Republic	General	MAP <70%	661	3	n.a.	Elective	n.a.
Juri et al. (56)	2018	Japan	General	MAP <65 mmHg	45	3	16 (35.6%)	Elective	General Surgery
Kalezic et al. (57)	2013	Serbia	General	SAP <80%	1,252	3	1,081 (86.3%)	Elective	General Surgery
Katori et al. (58)	2023	Japan	General	MAP <65 mmHg	11,210	4	5,457 (48.7%)	Both	Oral and maxillofacial Surgery
Kaydu et al. (59)	2018	Turkey	General	MAP <80%	80	3	38 (48.7%)	Elective	General Surgery
Kendale et al. (60)	2018	USA	General	MAP <55 mmHg	13,323	4	7,441 (56.0%)	n.a.	Gynecology
Khaled et al. (61)	2023	Egypt	General	MAP <80%	133	3	61 (45.9%)	Elective	n.a.
Kim et al. (62)	2022	South Korea	Spinal	SAP <90 mmHg and SAP <80%	50	3	10 (20.0%)	Elective	Urology
Klasen et al. (63)	2003	Germany	Spinal	MAP <70%	2,619	4	1,296 (49.5%)	Elective	Orthopedic Surgery
Kondo et al. (64)	2023	Japan	General	MAP <65 mmHg	261	4	57 (21.9%)	Elective	Urology
Kose et al. (65)	2012	Turkey	General	MAP <70%	157	3	75 (47.8%)	Elective	General Surgery
Lal et al. (66)	2023	India	Spinal	SAP <90 mmHg or SAP <80% or MAP <60 mmHg	75	3	17 (22.7%)	Elective	Orthopedic Surgery
Lee et al. (67)	2022	South Korea	General	Blood pressure <65 mmHg	888	3	486 (54.7%)	Elective	General Surgery
Lee et al. (68)	2024	South Korea	General	MAP <65 mmHg or MAP <80%	421	4	236 (56.1%)	Both	General Surgery
Li et al. (69)	2024	Taiwan	Spinal	SAP <90 mmHg	999	4	999 (100%)	Elective	Obstetrics
Lin et al. (70)	2011	Taiwan	General	SAP <90 mmHg or SAP <70%	1,311	4	830 (63.3%)	Both	n.a.
Maitra et al. (71)	2020	India	General	SAP <70% or MAP <80% or SAP <90 mmHg and MAP <65 mmHg	112	3	58 (51.8%)	Elective	n.a.
Malima et al. (72)	2019	South Africa	Spinal	SAP <100 mmHg or SAP <25%	357	3	212 (59.4%)	Both	Orthopedic Surgery
Mohammed et al. (73)	2021	India	General	MAP <65 mmHg or MAP <70%	110	2	52 (47.3%)	Elective	n.a.
Morisawa et al. (74)	2022	Japan	General	SAP <70 mmHg	142	4	57 (40.0%)	n.a.	Neurosurgery
Moschovaki et al. (75)	2023	Greece	Spinal	MAP ≤65 mmHg or MAP <75%	61	3	33 (54.1%)	n.a.	Orthopedic Surgery
Ni et al. (76)	2022	China	Spinal	MAP <60 mmHg or MAP <70%	90	3	42 (46.7%)	Elective	Urology
Oh et al. (77)	2024	South Korea	General	MAP <65 mmHg	157	3	90 (57.3%)	Elective	General Surgery
Ohpasanon et al. (78)	2008	Thailand	Spinal	SAP <100 mmHg and SAP <80%	807	3	807 (100%)	Both	Obstetrics
Okamura et al. (79)	2019	Japan	General	MAP <60 mmHg or MAP <70%	82	3	46 (56.1%)	Elective	n.a.
Saengrung et al. (80)	2022	Thailand	General	SAP <90 mmHg	83	4	22 (29.0%)	Emergency	Neurosurgery
Salama et al. (81)	2019	Egypt	Spinal	SAP <90 mmHg or SAP <70% or MAP <60 mmHg	100	3	55 (55.0%)	Elective	Orthopedic Surgery
Saranteas et al. (82)	2019	Greece	Spinal	MAP ≤65 mmHg or MAP <75%	70	3	41 (56.5%)	Elective	Orthopedic Surgery
Schonberger et al. (83)	2022	USA	General	MAP <55 mmHg	319,948	4	165,733 (51.8%)	Both	General Surgery
Shao et al. (84)	2022	China	General	MAP <65 mmHg or MAP <70%	61	3	29 (47.5%)	Elective	n.a.
Sharma et al. (85)	2024	India	General	MAP <65 mmHg or MAP <80%	100	3	65 (65.0%)	Elective	n.a.
Singh et al. (86)	2019	India	Spinal	MAP <65 mmHg or MAP <80%	40	3	40 (100%)	Elective	Obstetrics
Somboonviboon et al. (87)	2008	Thailand	Spinal	SAP <70%	722	3	722 (100%)	Both	Obstetrics
Südfeld et al.(88)	2017	Germany	General/Spinal	SAP <90 mmHg	2,037	4	901 (44.2%)	Both	n.a.
Taffe et al. (89)	2009	Switzerland	General/Regional	Blood pressure <70%	147,573	4	n.a.	Both	n.a.
Tarao et al. (90)	2021	Japan	General	MAP ≤50 mmHg	200	4	77 (33.5%)	Elective	n.a.
Thirunelli et al. (91)	2021	India	General	MAP <60 mmHg or MAP <80%	106	3	53 (50.0%)	Elective	n.a.
Walsh et al. (3)	2013	USA	n.a.	MAP <55 mmHg	33,330	4	16,836 (50.5%)	Both	n.a.
Wang et al. (92)	2022	China	General	MAP <65 mmHg or MAP <80%	99	3	34 (34.3%)	Elective	General Surgery
Wang et al. (93)	2024	China	General	SAP ≤80 mmHg or SBP <80% or MAP ≤60 mmHg	1,461	4	192 (23.9%)	n.a.	Cardiac Surgery
Yatabe et al. (94)	2020	Japan	General/Spinal	MAP <60 mmHg	172	4	45 (26.2%)	n.a.	Urology
Yilmaz et al. (95)	2022	Turkey	Spinal	SAP <90 mmHg or MAP <60 mmHg	95	3	33 (34.7%)	Elective	Orthopedic Surgery
Zhang et al. (96)	2016	China	General	MAP <60 mmHg or MAP <70%	90	3	47 (52.2%)	Elective	Cardiac Surgery

DAP, diastolic arterial pressure; IOH, intraoperative hypotension; LoE, level of evidence based on (97); MAP, mean arterial pressure; n.a., not available; SAP, systolic arterial pressure.

### Definition of IOH

The definition of IOH varied across studies (Table 2). In 25 studies, IOH was defined based on an absolute threshold for systolic arterial pressure (SAP) or mean arterial pressure (MAP). 14 studies defined IOH as a relative reduction in SAP or MAP compared to baseline values. In 36 studies, a combination of these criteria was used, whereas three studies described IOH as a general decrease in blood pressure.

### Patient characteristics

14 preoperative characteristics were identified and included in the analysis. These were then further classified into the following categories: patient-specific characteristics, pre-existing comorbidities, pre-existing medication, and emergency interventions. A meta-analysis was only feasible for the occurrence of IOH. For other outcomes—such as the rate, duration, and severity of IOH—the available data were too scarce and too heterogeneous to allow for meaningful synthesis.

**Figure 2:**
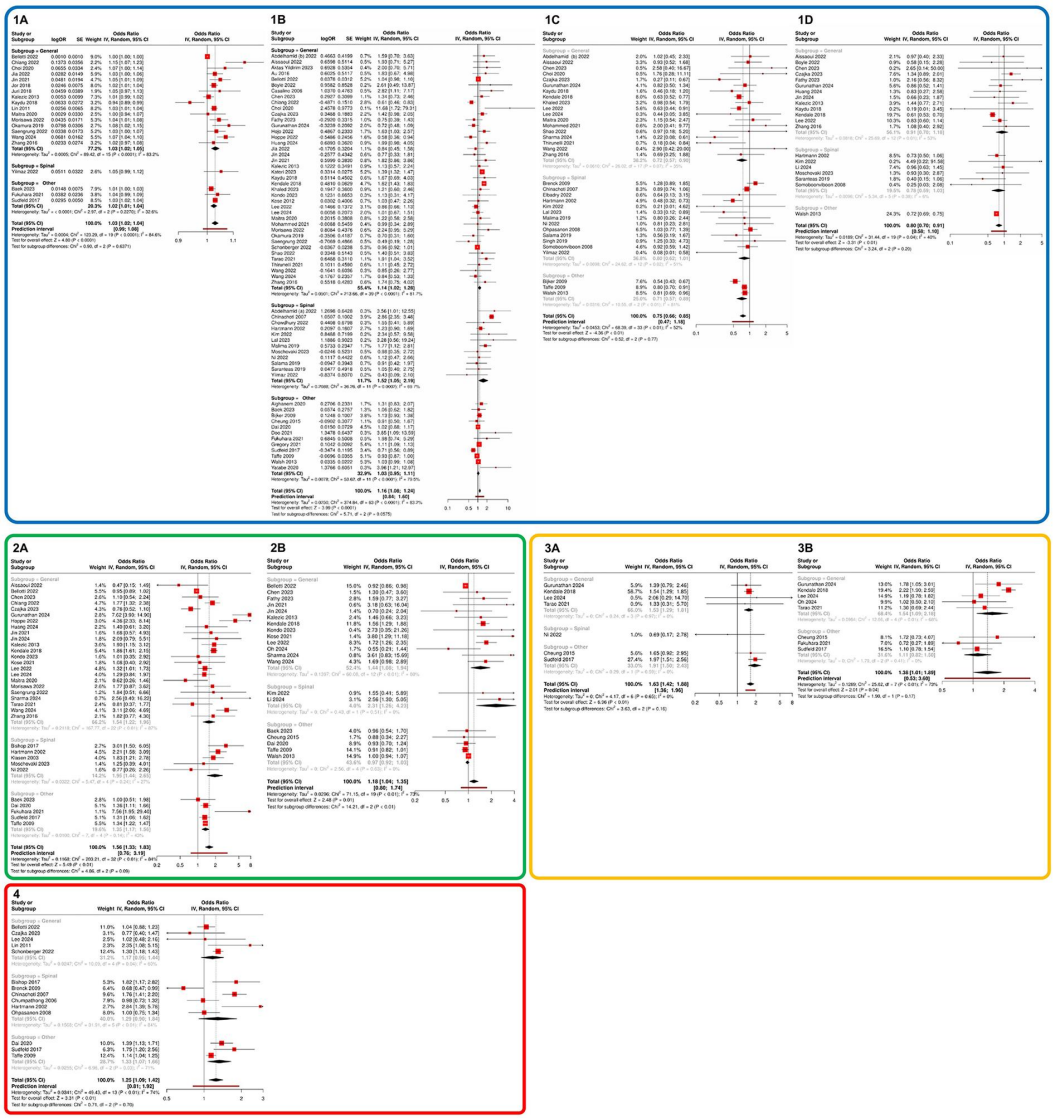
Meta-analysis of influencing factors regarding the probability of IOH occurrence. Section 1 (blue) presents specific patient characteristics: **(1A)** age, **(1B)** sex, **(1C)** ASA status I vs. II, and **(1D)** ASA status II vs. III. Section 2 (green) illustrates pre-existing comorbidities: **(2A)** arterial hypertension and **(2B)** diabetes mellitus. Section 3 (orange) depicts pre-existing medication: **(3A)** ACEI and **(3B)** ARB. Section 4 (red) highlights the impact of emergency surgery. Subgroups were defined as studies that exclusively investigated general anaesthesia, those that focused solely on regional anaesthesia, and studies that included both anaesthesia techniques or purely regional procedures. The latter were categorized under “Other”. ACEI, angiotensin-converting enzyme inhibitors; ARB, angiotensin II receptor blockers; ASA, American society of anesthesiologists; IOH, intraoperative hypotension.

In the patient-specific characteristics, increasing age was associated with a 3% higher risk of IOH (OR 1.03, 95% CI 1.02–1.04, 20 studies; see Figure 2(1A)]. This effect was found to be statistically significant in both the group that considered only general anesthesia (OR 1.03, 95% CI 1.02–1.05, 16 studies). Furthermore, female sex was found to be associated with a 16% increased risk of IOH [OR 1.16, 95% CI 1.08–1.24, 64 studies; see Figure 2[1B]). In the comparison of general anesthesia to spinal anesthesia, female sex was a significant influencing factor in the general anesthesia group (OR 1.14, 95% CI 1.02–1.28, 40 studies) as well as in the spinal anesthesia group (OR 1.52, 95% CI 1.05–2.19, 12 studies).

The assessment of preoperative health status based on ASA classification demonstrated a stepwise increase in IOH risk with higher ASA classes. Patients with ASA I had a 25% lower risk of IOH compared to patients with ASA II (OR 0.75, 95% CI 0.66–0.85; 34 studies; see Figure 2[1C]). Likewise, patients with ASA II had a 20% lower risk compared to patients with ASA III (OR 0.80, 95% CI 0.70–0.91; 20 studies; see Figure 2[1D]).

With regard to pre-existing comorbidities, patients with a known diagnosis of diabetes mellitus had an 18% higher likelihood of experiencing IOH (OR 1.18, 95% CI 1.04–1.35, 20 studies), whereas a history of hypertension was associated with a 56% increased probability of developing IOH (OR 1.56, 95% CI 1.33–1.83, 33 studies) (see Figures 2[2A/B]. These associations remained significant across all subgroup analyses. In contrast, body mass index (BMI) showed no significant impact on the risk of IOH (OR 1.00, 95% CI 0.99–1.02, 18 studies; see Supplementary Figure S1A).

The analysis of preoperative hemodynamic parameters revealed that SAP, MAP, and diastolic arterial pressure (DAP), as well as heart rate, had no significant influence on the risk of developing IOH (see Supplementary Figure S1). In contrast, the evaluation of preoperative long-term medication use indicated that the intake of ACEI was associated with a 63% increased probability of IOH (OR 1.63, 95% CI 1.42–1.88, 7 studies), whereas the use of ARBs was linked to a 38% increased likelihood of IOH (OR 1.38, 95% CI 1.01–1.89, 8 studies) (see Figure 2[3A,B]). Regarding the likelihood of IOH occurring during emergency surgeries, a significant increase of 25% was observed (OR 1.25, 95% CI 1.09–1.42, 14 studies; see Figure 2[4]).

**Figure 3:**
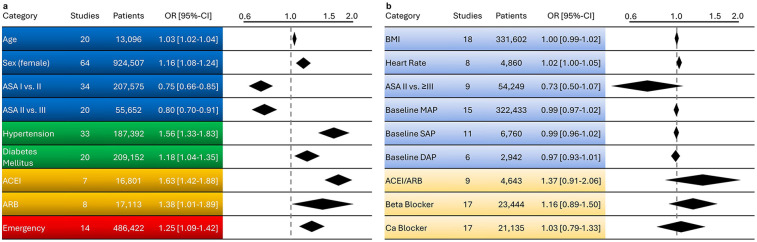
Overview of factors influencing the likelihood of intraoperative hypotension. In panel **(a)**, significant factors are shown in stronger colours, while in panel **(b)**, non-significant factors are displayed in lighter shades, each with their respective odds ratios. (Blue) represents specific patient characteristics, (green) illustrates pre-existing comorbidities, (orange) depicts pre-existing medication, and (red) highlights the impact of emergency surgery. ACEI, angiotensin-converting enzyme inhibitors; ARB, angiotensin II receptor blockers; ASA, American society of anesthesiologists; BMI, body mass index; Ca, calcium; CI, confidence Interval; OR, odds rat.

A comprehensive overview of all results, including the corresponding overall ORs, is presented in Figure 3.

### Heterogeneity and risk of bias

Overall heterogeneity was remarkably high, with an *I*^2^ > 75%. However, when examining the significant influencing factors ASA status II vs. III and ACEI, heterogeneity was moderate (*I*^2^ < 50%) and low (*I*^2^ = 0%), respectively (see Figure 2 and Supplementary Figure S1). Regarding publication bias, only a minor distortion of the published data was observed (see Supplementary Figure S2). The risk of bias was assessed as low to moderate in most studies; four prospective studies demonstrated a high risk of bias (see Supplementary Tables S2–S4).

## Discussion

Our systematic review confirmed that IOH remains inconsistently defined across the literature, with substantial variability in threshold values and measurement methods. This lack of a uniform definition was consistently reflected in the included studies and represents a central challenge in synthesizing evidence. Accordingly, in our analysis we adopted a definition-inclusive approach, retaining the original IOH definitions used by individual studies. This allowed us to investigate patientś susceptibility to IOH across real-world practice variation. Therefore, our results should be interpreted as reflecting vulnerability to intraoperative hypotensive episodes, rather than referencing a single fixed pressure threshold. Despite this heterogeneity, the meta-analysis identified several patient-related factors that are significantly associated with a higher likelihood of IOH, including increasing age, female sex, higher ASA classification, preexisting hypertension, diabetes mellitus, chronic use of ACEIs or ARBs, and emergency surgical procedures.

Despite its high clinical relevance, IOH remains without a universally accepted definition, which continues to impede both clinical standardization and scientific advancement. The variability in thresholds and diagnostic criteria limits comparability across studies and complicates the development of evidence-based treatment protocols. Notably, even major clinical guidelines reflect this lack of consensus. This inconsistency highlights the urgent need for a harmonized, evidence-based definition of IOH to enable coherent risk stratification, intraoperative decision-making, and outcome evaluation across clinical settings within an individual patient treatment approach (98, 99).

Intraoperative hemodynamics result from a complex interplay of systemic, pharmacological, surgical, and patient-specific factors and should therefore be understood as a multimodal concept. Rather than focusing on isolated variables, it is essential to consider the dynamic interaction of multiple risk components, which may collectively contribute to the onset and severity of IOH.

Several studies have demonstrated that IOH can lead to severe organ dysfunction (1–3, 100). In this context, the identification of individual patient preoperative risk factors may become a crucial first step targeting precision medicine. However, it still remains scarce which preoperative patient characteristics are associated with an increased risk of developing IOH. Dana et al., in their systematic review, investigated the role of preoperative ultrasound in predicting IOH and identified the preoperative inferior vena cava collapsibility index (IVC-CI) as a surrogate for volume responsiveness as a strong predictor of post-induction hypotension (10). However, other metanalysis doubt the usefulness of IVC evaluation at all (101). Importantly, IOH should be regarded not as a disease entity but as a clinical symptom indicative of diverse underlying intraoperative pathophysiological mechanisms (5). This heterogeneity necessitates a structured intraoperative diagnostic pathway to distinguish between different hemodynamic causes, such as vasodilation, hypovolemia, or myocardial depression, which can be conceptualized as distinct endotypes (6). Accurate intraoperative interpretation is thus essential for effective and individualized management. While MAP thresholds provide practical surrogate targets, they do not account for interindividual differences in vascular tone, autoregulatory capacity and pulse pressure propagation. In addition, postoperative complications are more closely related to impaired organ oxygen distribution/consumption relying on both adequate blood flow and arterial pressure according to the law of Ohm. Thus, future perioperative monitoring and clinical practice must incorporate more than only pressure-related targets to improve patientś outcome.

Our findings now contribute to a more comprehensive understanding of IOH susceptibility and support the use of structured preoperative risk stratification to identify vulnerable patients. It will be essential to adopt a patient-specific approach based on individual characteristics to develop and implement tailored therapeutic strategies aimed at preventing IOH more effectively (102). Such an approach enables individualized intraoperative management and may ultimately improve postoperative outcomes.

Chen et al. were among the first to systematically explore patient characteristics in relation to IOH, suggesting that older age, female sex, antihypertensive medication use, and emergency procedures may increase IOH risk (103). However, their conclusions were based solely on descriptive data from 12 included studies, without conducting a meta-analysis. In contrast, our review included 78 studies, as we applied a broader search strategy and were additionally able to incorporate results from studies using patient-level data, thereby enabling, for the first time, a robust meta-analysis within a large and heterogeneous surgical patient cohort. Our findings corroborate the initial hypotheses by Chen et al., confirming significant associations between IOH and the above-mentioned factors.

Bos et al. also examined age and sex as risk factors in their systematic review (11). While they did not find a significant influence of female sex on IOH exposure in the general cohort (OR 1.10, 95% CI 0.98–1.23), a subset analysis of studies with an average age ≥65 years showed increased IOH exposure in females (OR 1.17, 95% CI 1.01–1.35). Similarly, in our analysis, female sex was associated with a 16% higher likelihood of experiencing IOH. Prior studies have emphasized the need to consider sex-specific factors in clinical decision-making (104, 105). It has been demonstrated that, even under the same therapeutic regimen, female sex is an independent risk factor for increased mortality and that different safety cut-offs may be necessary (105). Whether comparable safety cut-offs for IOH are adequate among male and female patients remains unknown and should be investigated in the future. Linked to this, the sex of the anesthesia provider may play a role in addition to the patient's sex: a recent study demonstrated that female anesthesia providers more effectively prevented IOH, intraoperative desaturation, and hyper- or hypocapnia (106).

Our data identified patients with preexisting arterial hypertension and chronic use of Renin-Angiotensin-Aldosterone System (RAAS)–modulating medications (e.g., ACEIs) as particularly vulnerable, with a more than 50% higher probability of IOH. Duceppe et al. reported that in patients undergoing major vascular surgery, long-term antihypertensive therapy was independently associated with increased risk of postoperative AKI (107). On the contrary, others stated that current evidence is insufficient to recommend routine discontinuation of ACEIs/ARBs on the day of surgery but stressed the need for anesthesiologists to remain vigilant for IOH and manage it proactively (108, 109). Discontinuation has also been found to increase the likelihood of clinical significant hypertension in non-cardiac surgery (110). Moreover, it is important to note that the influence of surgical extent on the risk of IOH has not yet been adequately addressed, even in randomized controlled trials (7). While several of these associations have been reported previously, the available evidence has remained fragmented. Importantly, our findings confirm the physiologically expected increased susceptibility to IOH in patients with chronic hypertension, which is likely related to impaired baroreflex sensitivity and altered vascular compliance, and refine the magnitude of this association through pooled effect estimation. The novelty of our analysis lies in aggregating and meta-analytically quantifying these associations across 78 worl-wide studies and more than 930,000 patients. Moreover, by evaluating hypertensive disease and chronic RAAS-modulating medication use separately, our results suggest that these represent related but distinct contributors to IOH risk. Demonstrating that these effects are consistent across diverse anesthesia techniques and surgical specialties strengthens their validity as preoperative risk surrogates and supports their use in a structured, individualized perioperative hemodynamic management.

### Limitations

One of the key strengths and primary objectives of our study is its comprehensive inclusion of the wide variability in IOH definitions present in the literature. Though this heterogeneity has already been suggested (4), our systematic review now provides a unique and thorough meta-analytical synthesis that captures the full spectrum of IOH definitions used up to date. While this complexity presents challenges, it also represents an important advance by reflecting real-world variability and enhancing the generalizability of our findings. To account for between-study differences, we applied a random-effects model, yielding more conservative and broadly applicable effect estimates. Importantly, despite the diversity, most studies employed comparable thresholds for absolute or relative blood pressure reductions, supporting the overall interpretability of the results.

Additionally, our analysis included both cardiac and non-cardiac surgery populations. Given the distinct hemodynamic profiles and perioperative management strategies between these groups, this broad inclusion adds another layer of real-world heterogeneity, which in turn might be considered a further strength by capturing a wider clinical spectrum and improving external validity.

A notable limitation of our meta-analysis is the heterogeneity and incomplete reporting of key clinical context variables across studies. In particular, type of surgery, intraoperative medication strategies (e.g., vasopressors, vasodilators, beta-blockers), and patient-specific pathophysiological profiles (e.g., trauma, frailty, myelopathy) were reported inconsistently or aggregated into broad categories, which did not allow for statistically robust subgroup analyses. Therefore, the associations identified in this review should be interpreted as reflecting general patientś susceptibility to IOH rather than interactions between patient characteristics and specific surgical or pharmacologic management strategies, especially in the context of underlying cardiovascular comorbidity. Moreover, considerable variability in study inclusion criteria and frequent underreporting of clinically relevant subgroups (e.g., patients with heart failure or atrial fibrillation) may limit the generalizability of our findings and introduce selection bias.

Beyond commonly assessed factors like age, sex, and comorbidities, functional status has been identified in previous research as a significant predictor of IOH. Specifically, preoperative frailty or functional impairment independently increases IOH risk, even after adjusting for age and ASA classification (9). Unfortunately, due to inconsistent reporting, this important parameter could not be included in our quantitative synthesis. Similarly, certain high-risk populations remain underrepresented in current literature. Future investigations, such as the ongoing PeriopCAreHF trial, are expected to address these gaps, thereby refining patient stratification and perioperative management strategies (111).

Due to considerable heterogeneity and insufficient reporting of IOH episode duration and severity across studies, we were unable to incorporate these important parameters within a time-weighted area under the curve approach into our analysis and were therefore restricted to only a binary consideration of IOH occurrence. Yet, existing studies suggest that the duration and depth of IOH episodes play a pivotal role in the development of organ dysfunction (3). Bijker et al. demonstrated that the more severe the hypotension, the shorter the threshold duration for a significant increase in mortality (2). Although heterogeneity was primarily driven by differences in IOH definitions, additional variability in effect estimates due to studies with mixed or regional anesthesia approaches (categorized as “Other” in the Methods section) cannot be excluded. Finally, the hemodynamic influence of neuraxial techniques such as thoracic epidural anesthesia (TEA) could not be analyzed separately due to limited subgroup data; future studies should therefore evaluate TEA and combined general–epidural approaches as distinct anesthetic strategies with potentially unique IOH risk profiles.

As the evidence base expands, meta-regression techniques could help systematically explore potential effect modifiers. Further research is also needed to investigate whether the patient-related risk factors identified in our analysis not only influence the incidence but also the duration and severity of IOH. Incorporating these aspects could enhance perioperative risk stratification and support more targeted hemodynamic management strategies.

## Conclusion

Our analysis underscores the considerable variability in definitions of IOH across studies, which complicates comparisons and the interpretation of findings. Establishing standardized IOH definitions in the future is crucial to enable more consistent research and improve clinical decision-making. Despite this variability, we identified a significant impact of patient characteristics, such as age, sex, comorbidities, and chronic medication use, on the incidence of IOH. However, further research is needed to explore how these factors may also influence the duration and severity of IOH episodes. To guide future clinical implementation and research, a standardized and physiologically informed definition of IOH are needed that allow distinguishing different mechanisms of intraoperative hypotensive episodes.

## Data Availability

The original contributions presented in the study are included in the article/Supplementary Material, further inquiries can be directed to the corresponding author.

## Supplementary material

Supplementary File 1 Visual abstract. Created in BioRender. Markus, M. (2025), https://BioRender.com/a6gg90v.
