# Supplementary material for: Preoperative patient risk factors for intraoperative hypotension: a systematic review and meta-analysis
Source: Front Cardiovasc Med. 2025 Dec 5;12:1709004. doi: 10.3389/fcvm.2025.1709004 (PMC12714980; doi:10.3389/fcvm.2025.1709004)
Supplement: Supplementary file 1 [file Datasheet1.pdf]

Supplementary Appendix

Preoperative Patient Risk Factors for Intraoperative Hypotension: A Systematic Review and Meta-Analysis

Nils Daum[1,2], Daniel Bill[1], Moritz Thiele[2], Julian Felber[2], Dario von Wedel[2], Claudia Spies[1], Felix Balzer[2], Rudolf Mörgeli[1], Oliver Hunsicker[1], Anika Müller[1], Dennis Contag[1], Anne Pohrt[3], Annika Bald[1], Max Kayser[1], Sascha Treskatsch[4], Maximilian Markus[1*]

[1] Charité – Universitätsmedizin Berlin, Corporate Member of Freie Universität Berlin and Humboldt Universität zu Berlin, Department of Anaesthesiology and Intensive Care Medicine (CCM/CVK), Berlin, Germany

[2] Charité – Universitätsmedizin Berlin, Corporate Member of Freie Universität Berlin and Humboldt Universität zu Berlin, Institute of Medical Informatics, Berlin, Germany

[3] Charité – Universitätsmedizin Berlin, Corporate Member of Freie Universität Berlin and Humboldt Universität zu Berlin, Institute for Biometry and Clinical Epidemiology, Berlin, Germany

[4] Charité – Universitätsmedizin Berlin, Corporate Member of Freie Universität Berlin and Humboldt Universität zu Berlin, Department of Anaesthesiology and Intensive Care Medicine (CBF), Berlin, Germany

***Corresponding author:**

Dr. med. Maximilian Markus
Charité – Universitätsmedizin Berlin
Charitéplatz 1
10117 Berlin
Germany
maximilian.markus@charite.de

Table S1. Systematic search strategy for the databases used in each case.

**Embase and MEDLINE** (via Ovid)	Search #1 (Surgery)	((((“General Surgery”) OR (“Surgical Procedures”) OR (“Intraoperative Complications”) OR (Anesthesia) OR (Anesthesiology)).sh. OR ((Surgery) OR (Operative) OR (Surgical) OR (Perioperative) OR (Intraoperative) OR (Operative) OR (Postoperative) OR (Anesthesia*) OR (Anesthesiolog*)).ab. OR ((Surgery) OR (Operative) OR (Surgical) OR (Perioperative) OR (Intraoperative) OR (Operative) OR (Postoperative) OR (Anesthesia*) OR (Anesthesiolog*)).ti.)
	Search #2 (Risk)	(("Risk").sh. OR (“Risk*”).ab. OR (“Risk*”).ti.)
	Search #3 (Hypotension)	(((Hypotension)).sh. OR ((“Low Blood Pressure”) OR (Hypotens*)).ab. OR ((“Low Blood Pressure”) OR (Hypotens*)).ti.)
	Final Search #1 AND #2 AND #3	
**Cochrane Library**	Search #1 (Surgery)	Mesh: General Surgery, Surgical Procedures, Intraoperative Complications, Anesthesia, Anesthesiology OR Title, Abstract, Keywords: Surgery, Operative, Surgical, Perioperative, Intraoperative, Operative, Postoperative, Anesthesia*, Anesthesiolog*
	Search #2 (Risk)	Mesh: Risk OR Title, Abstract, Keywords: Risk*
	Search #3 (Hypotension)	Mesh: Hypotension OR Title, Abstract, Keywords: Low Blood Pressure
	Final Search #1 AND #2 AND #3	

Table S2. Risk of Bias Assessment using the RoB-2 Tool for Randomized Controlled Studies

Author	Year	D1	D2	D3	D4	D5	Overall
Mohammed	2021	!	+	+	+	!	!

D1 Randomization process; D2 Deviations from intended interventions; D3 Missing outcome data; D4 Measurement of the outcome; D5 Selection of the reported result; + Low risk; ! Some concerns

Table S3. Risk of Bias Assessment using the ROBINS-E Tool for Prospective Non-randomized Studies

Author	Year	D1	D2	D3	D4	D5	D6	D7	Overall
Sharma	2024	Low	Low	Low	Low	Low	Some concerns	Low	**Low**
Abdelhamid (a)	2022	Low	Low	Low	Low	Low	Low	Low	**Low**
Abdelhamid (b)	2022	Some concerns	Low	Low	Low	Low	Low	Low	**Low**
Aissaoui	2022	Some concerns	Low	Low	Low	Low	Some concerns	Low	**Some concerns**
Aktas Yildirim	2023	Low	Low	Low	Low	Low	Some concerns	Low	**Low**
Au	2016	Some concerns	Low	Low	Low	Low	Some concerns	Low	**Some concerns**
Bijker	2009	Low	Low	Low	Low	Low	Some concerns	Low	**Low**
Bishop	2017	Some concerns	Low	Some concerns	Low	Low	Some concerns	Low	**Some concerns**
Boyle	2022	Some concerns	Low	Low	Low	Low	Some concerns	Low	**Some concerns**
Chen	2023	Low	Low	Low	Low	Low	Some concerns	Low	**Low**
Cheung	2015	High	Low	Low	Low	Low	Some concerns	Low	**High**
Chinachoti	2007	Some concerns	Low	Low	Low	Low	Low	Low	**Low**
Choi	2020	Low	Low	Low	Low	Low	Some concerns	Low	**Low**
Chowdhury	2022	Low	Low	Low	Low	Some concerns	Low	Low	**Low**
Czajka	2023	Low	Low	Low	Low	Low	Some concerns	Low	**Low**
Elbadry	2022	Low	Low	Some concerns	Low	Low	Some concerns	Low	**Some concerns**
Fathy	2023	Some concerns	Low	Low	Low	Low	Some concerns	Low	**Some concerns**
Gurunathan	2024	Low	Low	Low	Low	Low	Some concerns	Low	**Low**
Hoppe	2022	Low	Low	Low	Low	Low	Low	Low	**Low**
Huang	2024	Low	Low	Low	Low	Low	Some concerns	Low	**Low**
Jin	2024	Low	Low	Low	Low	Low	Some concerns	Low	**Low**
Jor	2018	Low	Low	Low	Low	High	Low	Low	**High**
Juri	2018	Low	Low	Low	Low	Low	Some concerns	Low	**Low**
Kalezic	2013	Low	Low	Some concerns	Low	Low	Low	Low	**Low**
Kaydu	2018	Low	Low	Low	Low	Low	Low	Low	**Low**
Khaled	2023	Low	Low	Low	Low	Low	Low	Low	**Low**
Kim	2022	Low	Low	Low	Low	Low	Some concerns	Low	**Low**
Kose	2012	Low	Low	Low	Low	Low	Low	Low	**Low**

Lal	2023	Low	Low	Low	Low	Low	Some concerns	Low	**Low**
Lee	2022	Low	Low	Low	Low	Low	Some concerns	Low	**Low**
Maitra	2020	Some concerns	Low	Low	Low	Low	Some concerns	Low	**Some concerns**
Malima	2019	Low	Low	Low	Low	Low	Some concerns	Low	**Low**
Moschovaki	2023	Low	Low	Low	Low	Low	Some concerns	Low	**Low**
Ni	2022	Low	Low	Low	Low	Low	Some concerns	Low	**Low**
Oh	2024	Low	Low	Low	Low	Some concerns	Some concerns	Low	**Some concerns**
Ohpasanon	2008	Low	Low	Some concerns	Low	Low	Some concerns	Low	**Some concerns**
Okamura	2019	Low	Low	Low	Low	Some concerns	Some concerns	Low	**Some concerns**
Salama	2019	Low	Low	Low	Low	Low	Some concerns	Low	**Low**
Saranteas	2019	Some concerns	Low	Some concerns	Low	Low	Some concerns	Low	**High**
Shao	2022	Low	Low	Low	Low	Low	Some concerns	Low	**Low**
Singh	2019	Some concerns	Low	Some concerns	Low	Low	Some concerns	Low	**High**
Somoboonviboon	2008	Low	Low	Some concerns	Low	Low	Low	Low	**Low**
Thirunelli	2021	Some concerns	Low	Low	Low	Low	Some concerns	Low	**Some concerns**
Wang	2022	Low	Low	Low	Low	Low	Some concerns	Low	**Low**
Yilmaz	2022	Low	Low	Low	Low	Low	Some concerns	Low	**Low**
Zhang	2016	Low	Low	Low	Low	Low	Some concerns	Low	**Low**

D1 Confounding; D2 Selection of participants; D3 Classification of exposure; D4 Deviations from intended exposure; D5 Missing data; D6 Measurement of outcomes; D7 Selection of the reported result

Table S4. Risk of Bias Assessment using the Newcastle-Ottawa Scale for Retrospective Non-randomized Studies

Author	Year	D1	D2	D3	D4	D5	D6	D7	D8	Overall
Alghanem	2020	b	a	a	a	a	a	a	a	**Some concerns**
Baek	2023	a	a	a	b	a b	a	a	a	**Some concerns**
Bellotti	2022	c	a	a	b	a b	a	a	a	**High risk**
Brenck	2009	b	a	a	b	a b	a	a	a	**Some concerns**
Casalino	2006	c	a	a	b	a	a	a	a	**High risk**
Chiang	2022	a	a	a	b	a b	a	a	a	**Some concerns**
Chumpathong	2006	b	a	a	b	a	a	a	a	**Some concerns**
Dai	2020	a	a	a	b	a b	a	a	a	**Some concerns**
Doo	2021	a	a	a	b	a b	a	a	a	**Some concerns**
Fukuhara	2021	a	a	a	b	a b	a	a	a	**Some concerns**
Gregory	2021	a	a	a	b	a b	a	a	a	**Some concerns**
Hartmann	2002	a	a	a	a	a	a	a	a	**Low risk**
Hojo	2022	a	a	a	b	a b	a	a	a	**Some concerns**
Jia	2022	a	a	a	a	a b	a	a	a	**Some concerns**
Jin	2021	a	a	a	b	a b	a	a	a	**Some concerns**
Katori	2023	a	a	a	b	a	a	a	a	**Some concerns**
Kendale	2018	a	a	a	b	a	a	a	a	**Some concerns**
Klasen	2003	a	a	a	a	a	a	a	a	**Low risk**
Kondo	2023	a	a	a	b	a b	a	a	a	**Some concerns**
Lee	2024	b	a	a	b	a b	a	a	a	**Some concerns**
Li	2024	b	a	a	b	a b	a	a	a	**Some concerns**
Lin	2011	a	a	a	b	a b	a	a	a	**Some concerns**
Morisawa	2022	a	a	a	b	a b	a	a	a	**Some concerns**
Saengrung	2022	b	a	a	b	a b	a	a	a	**Some concerns**
Schonberger	2022	a	a	a	b	a b	a	a	a	**Some concerns**
Südfeld	2017	a	a	a	b	a b	a	a	a	**Some concerns**
Taffe	2009	a	a	a	a	a b	a	a	a	**Some concerns**

Tarao	2021	a	a	a	b	a b	a	a	a	**Some concerns**
Walsh	2013	a	a	a	b	a b	a	a	a	**Some concerns**
Wang	2024	c	a	a	b	a b	a	a	a	**High risk**
Yatabe	2020	a	a	a	b	a b	a	a	a	**Some concerns**

D1 Representativeness of the exposed cohort; D2 Selection of the non-exposed cohort; D3 Ascertainment of exposure; D4 Demonstration that outcome was not present at start; D5 Adjustment for confounders; D6 Assessment of outcome; D7 Follow-up duration adequate; D8 Adequacy of follow-up / completeness of data; a low risk; b some concerns; c high risk

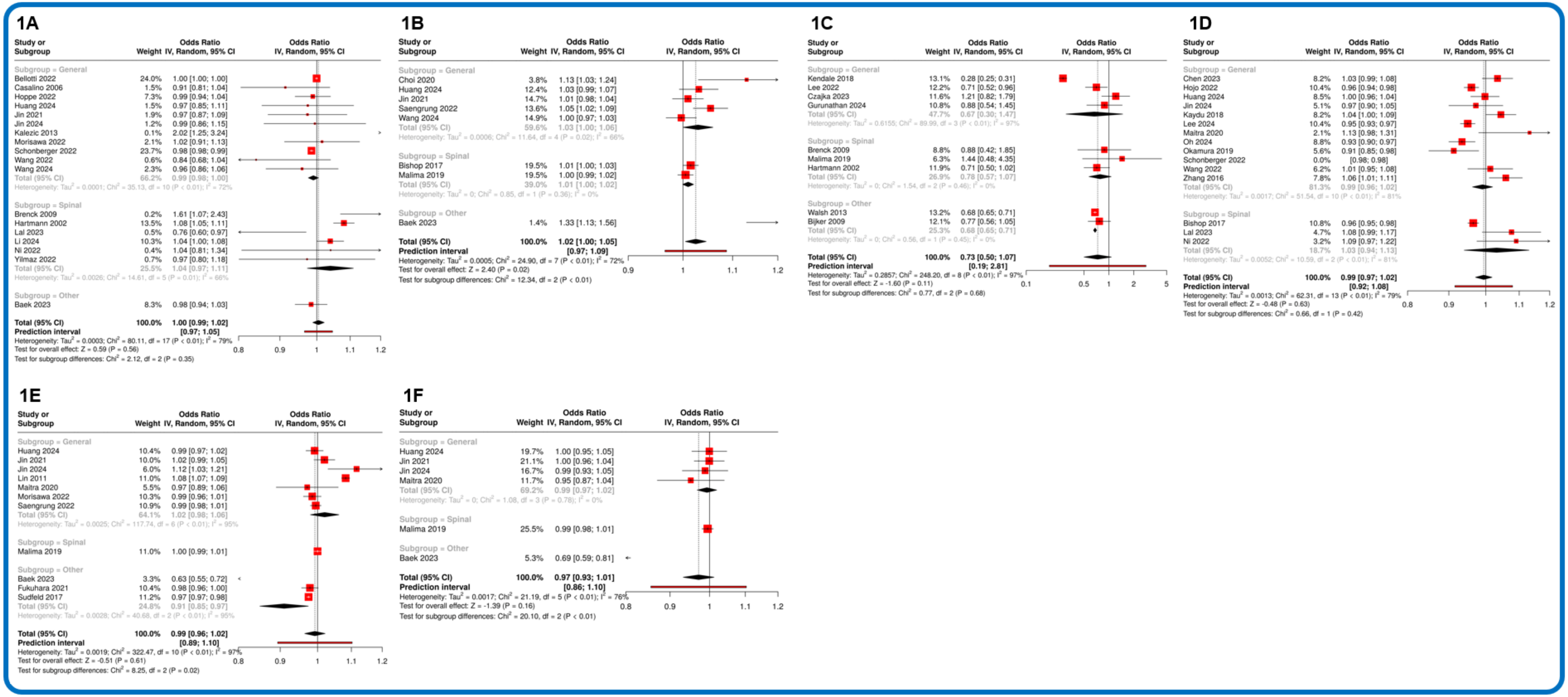
1A
Study or Subgroup | Weight | Odds Ratio IV, Random, 95% CI | Odds Ratio IV, Random, 95% CI
Subgroup = General
Bellotti 2022 24.0% 1.00 [1.00; 1.00]
Casalino 2006 1.5% 0.91 [0.81; 1.04]
Hoppe 2022 7.3% 0.99 [0.94; 1.04]
Huang 2024 1.5% 0.97 [0.85; 1.11]
Jin 2021 1.9% 0.97 [0.87; 1.09]
Jin 2024 1.2% 0.99 [0.86; 1.15]
Kalezic 2013 0.1% 2.02 [1.25; 3.24]
Morisawa 2022 2.1% 1.02 [0.91; 1.13]
Schonberger 2022 23.7% 0.98 [0.98; 0.99]
Wang 2022 0.6% 0.84 [0.68; 1.04]
Wang 2024 2.3% 0.96 [0.86; 1.06]
Total (95% CI) 66.2% 0.99 [0.98; 1.00]
Subgroup = Spinal
Brenck 2009 0.2% 1.61 [1.07; 2.43]
Hartmann 2002 13.5% 1.08 [1.05; 1.11]
Lal 2023 0.5% 0.76 [0.60; 0.97]
Li 2024 10.3% 1.04 [1.00; 1.08]
Ni 2022 0.4% 1.04 [0.81; 1.34]
Yilmaz 2022 0.7% 0.97 [0.80; 1.18]
Total (95% CI) 25.5% 1.04 [0.97; 1.11]
Subgroup = Other
Baek 2023 8.3% 0.98 [0.94; 1.03]
Total (95% CI) 100.0% 1.00 [0.99; 1.02]
Prediction interval [0.97; 1.05]
Test for overall effect: Z = 0.59 (P = 0.56)
Test for subgroup differences: Chi2 = 2.12, df = 2 (P = 0.35)
0.8 0.9 1 1.1 1.2
1B
Study or Subgroup | Weight | Odds Ratio IV, Random, 95% CI | Odds Ratio IV, Random, 95% CI
Subgroup = General
Choi 2020 3.8% 1.13 [1.03; 1.24]
Huang 2024 12.4% 1.03 [0.99; 1.07]
Jin 2021 14.7% 1.01 [0.98; 1.04]
Saengrung 2022 13.6% 1.05 [1.02; 1.09]
Wang 2024 14.9% 1.00 [0.97; 1.03]
Total (95% CI) 59.6% 1.03 [1.00; 1.06]
Subgroup = Spinal
Bishop 2017 19.5% 1.01 [1.00; 1.03]
Malima 2019 19.5% 1.00 [0.99; 1.02]
Total (95% CI) 39.0% 1.01 [1.00; 1.02]
Subgroup = Other
Baek 2023 1.4% 1.33 [1.13; 1.56]
Total (95% CI) 100.0% 1.02 [1.00; 1.05]
Prediction interval [0.97; 1.09]
Test for overall effect: Z = 2.40 (P = 0.02)
Test for subgroup differences: Chi2 = 12.34, df = 2 (P < 0.01)
0.9 1 1.1 1.2
1C
Study or Subgroup | Weight | Odds Ratio IV, Random, 95% CI | Odds Ratio IV, Random, 95% CI
Subgroup = General
Kendale 2018 13.1% 0.28 [0.25; 0.31]
Lee 2022 12.2% 0.71 [0.52; 0.96]
Czajka 2023 11.6% 1.21 [0.82; 1.79]
Gurunathan 2024 10.8% 0.88 [0.54; 1.45]
Total (95% CI) 47.7% 0.67 [0.30; 1.47]
Subgroup = Spinal
Brenck 2009 8.8% 0.88 [0.42; 1.85]
Malima 2019 6.3% 1.44 [0.48; 4.35]
Hartmann 2002 11.9% 0.71 [0.50; 1.02]
Total (95% CI) 26.9% 0.78 [0.57; 1.07]
Subgroup = Other
Walsh 2013 13.2% 0.68 [0.65; 0.71]
Bijker 2009 12.1% 0.77 [0.56; 1.05]
Total (95% CI) 25.3% 0.68 [0.65; 0.71]
Total (95% CI) 100.0% 0.73 [0.50; 1.07]
Prediction interval [0.19; 2.81]
Test for overall effect: Z = -1.60 (P = 0.11)
Test for subgroup differences: Chi2 = 0.77, df = 2 (P = 0.68)
0.1 0.5 1 2 5
1D
Study or Subgroup | Weight | Odds Ratio IV, Random, 95% CI | Odds Ratio IV, Random, 95% CI
Subgroup = General
Chen 2023 8.2% 1.03 [0.99; 1.08]
Hojo 2022 10.4% 0.96 [0.94; 0.98]
Huang 2024 8.5% 1.00 [0.96; 1.04]
Jin 2024 5.1% 0.97 [0.90; 1.05]
Kaydu 2018 8.2% 1.04 [1.00; 1.09]
Lee 2024 10.4% 0.95 [0.93; 0.97]
Maitra 2020 2.1% 1.13 [0.98; 1.31]
Oh 2024 8.8% 0.93 [0.90; 0.97]
Okamura 2019 5.6% 0.91 [0.85; 0.98]
Schonberger 2022 0.0% [0.98; 0.98]
Wang 2022 6.2% 1.01 [0.95; 1.08]
Zhang 2016 7.8% 1.06 [1.01; 1.11]
Total (95% CI) 81.3% 0.99 [0.96; 1.02]
Subgroup = Spinal
Bishop 2017 10.8% 0.96 [0.95; 0.98]
Lal 2023 4.7% 1.08 [0.99; 1.17]
Ni 2022 3.2% 1.09 [0.97; 1.22]
Total (95% CI) 18.7% 1.03 [0.94; 1.13]
Total (95% CI) 100.0% 0.99 [0.97; 1.02]
Prediction interval [0.92; 1.08]
Test for overall effect: Z = -0.48 (P = 0.63)
Test for subgroup differences: Chi2 = 0.66, df = 1 (P = 0.42)
0.8 0.9 1 1.1 1.2
1E
Study or Subgroup | Weight | Odds Ratio IV, Random, 95% CI | Odds Ratio IV, Random, 95% CI
Subgroup = General
Huang 2024 10.4% 0.99 [0.97; 1.02]
Jin 2021 10.0% 1.02 [0.99; 1.05]
Jin 2024 6.0% 1.12 [1.03; 1.21]
Lin 2011 11.0% 1.08 [1.07; 1.09]
Maitra 2020 5.5% 0.97 [0.89; 1.06]
Morisawa 2022 10.3% 0.99 [0.96; 1.01]
Saengrung 2022 10.9% 0.99 [0.98; 1.01]
Total (95% CI) 64.1% 1.02 [0.98; 1.06]
Subgroup = Spinal
Malima 2019 11.0% 1.00 [0.99; 1.01]
Subgroup = Other
Baek 2023 3.3% 0.63 [0.55; 0.72]
Fukuhara 2021 10.4% 0.98 [0.96; 1.00]
Sudfeld 2017 11.2% 0.97 [0.97; 0.98]
Total (95% CI) 24.8% 0.91 [0.85; 0.97]
Total (95% CI) 100.0% 0.99 [0.96; 1.02]
Prediction interval [0.89; 1.10]
Test for overall effect: Z = -0.51 (P = 0.61)
Test for subgroup differences: Chi2 = 8.25, df = 2 (P = 0.02)
0.8 0.9 1 1.1 1.2
1F
Study or Subgroup | Weight | Odds Ratio IV, Random, 95% CI | Odds Ratio IV, Random, 95% CI
Subgroup = General
Huang 2024 19.7% 1.00 [0.95; 1.05]
Jin 2021 21.1% 1.00 [0.96; 1.04]
Jin 2024 16.7% 0.99 [0.93; 1.05]
Maitra 2020 11.7% 0.95 [0.87; 1.04]
Total (95% CI) 69.2% 0.99 [0.97; 1.02]
Subgroup = Spinal
Malima 2019 25.5% 0.99 [0.98; 1.01]
Subgroup = Other
Baek 2023 5.3% 0.69 [0.59; 0.81]
Total (95% CI) 100.0% 0.97 [0.93; 1.01]
Prediction interval [0.86; 1.10]
Test for overall effect: Z = -1.39 (P = 0.16)
Test for subgroup differences: Chi2 = 20.10, df = 2 (P < 0.01)
0.8 0.9 1 1.1 1.2

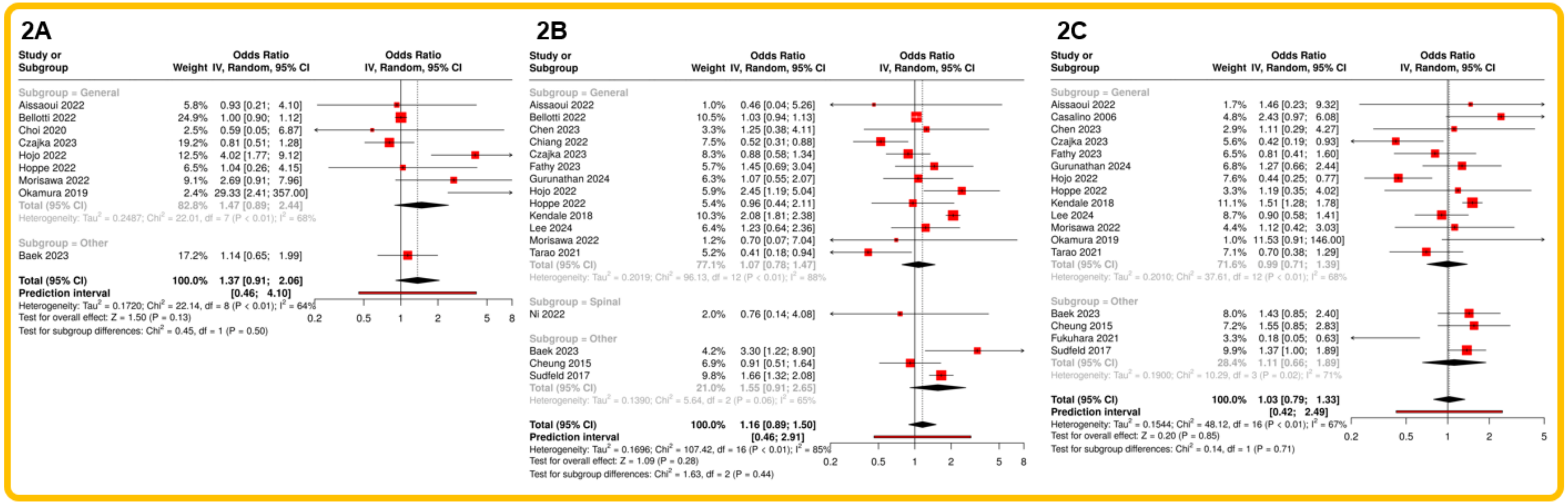

Figure S1. Meta-analysis of non-significant influencing factors regarding the probability of IOH occurrence.

Section 1 (blue) presents specific patient characteristics: (1A) BMI, (1B) ASA status II vs. ≥III, (1C) heart rate, (1D) baseline MAP, (1E) baseline SAP, (1F) baseline DAP. Section 2 (orange) depicts pre-existing medication: (2A) ACEI/ARB, (2B) beta blocker, (2C) Ca Blocker. Section 4 (red) highlights the impact of emergency surgery. Subgroups were defined as studies that exclusively investigated general anesthesia, those that focused solely on regional anaesthesia, and studies that included both anaesthesia techniques or purely regional procedures. The latter were categorized under "Other."

ACEI angiotensin-converting enzyme inhibitors; ARB angiotensin II receptor blockers; ASA American Society of Anesthesiologists; BMI Body Mass Index; Ca Calcium; DAP diastolic arterial pressure; IOH intraoperative hypotension; MAP mean arterial pressure; SAP systolic arterial pressure

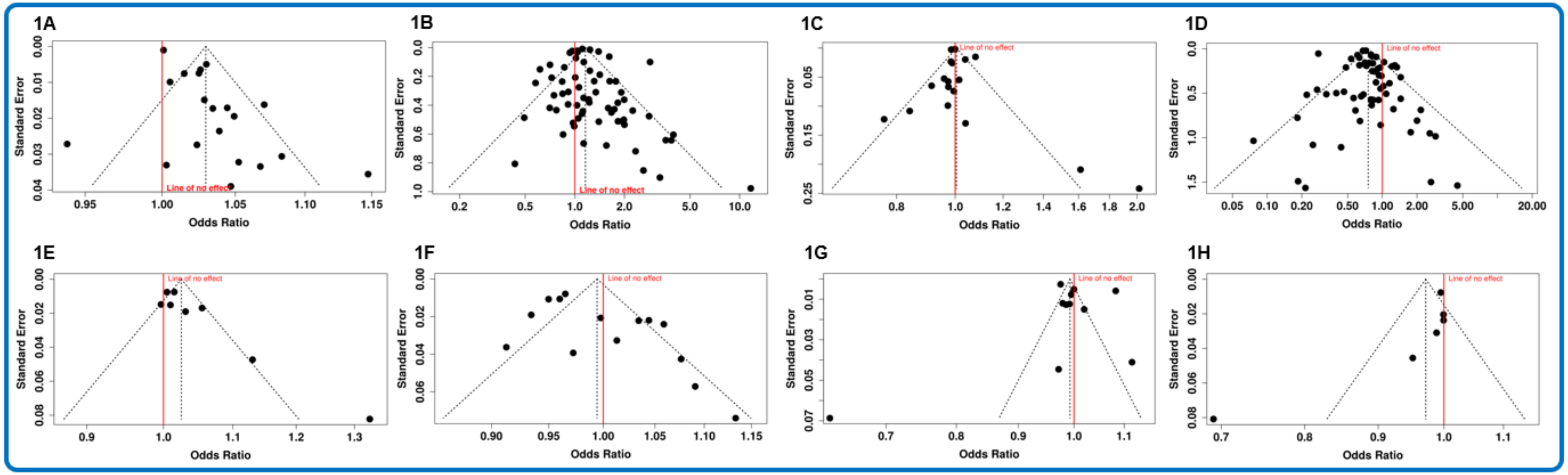

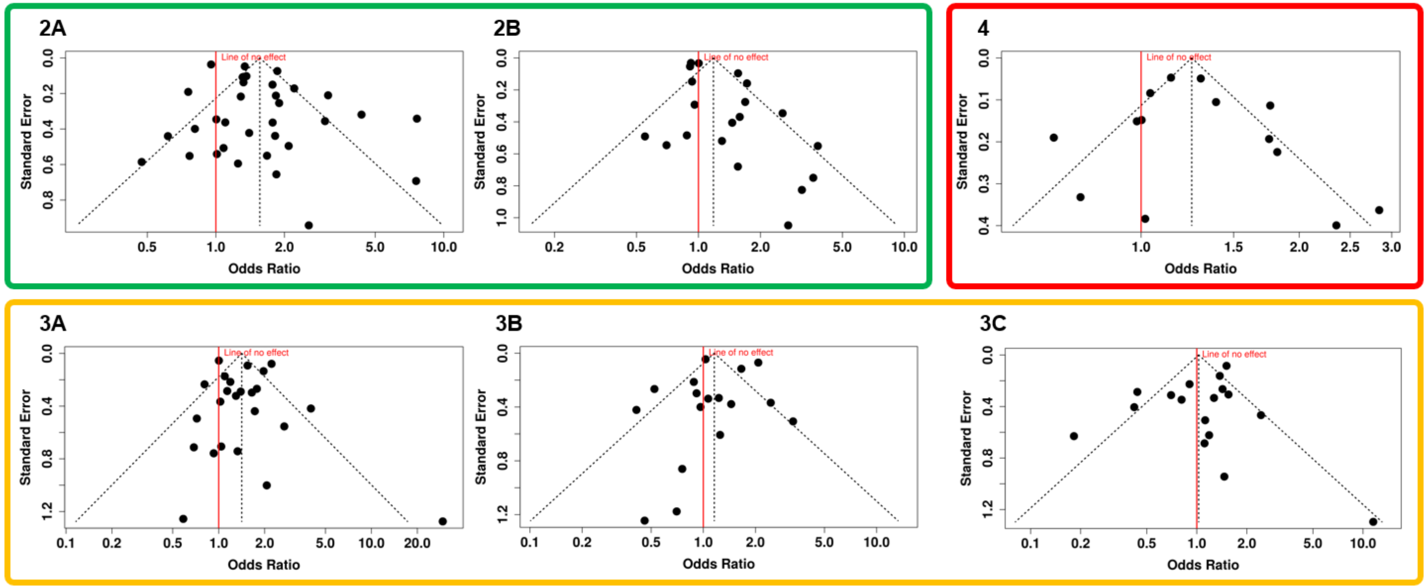

Figure S2. Funnel Plots

Section 1 (blue) presents specific patient characteristics: (1A) age, (1B) sex, (1C) BMI, (1D) ASA status, (1E) heart rate, (1F) baseline MAP, (1G) baseline SAP, (1H) baseline DAP. Section 2 (green) illustrates pre-existing comorbidities: (2A) arterial hypertension and (2B) diabetes mellitus. Section 3 (orange) depicts pre-existing medication: (3A) ACEI/ARB, (3B) beta blocker, (3C) Ca Blocker. Section 4 (red) highlights the impact of emergency surgery.

ACEI angiotensin-converting enzyme inhibitors; ARB angiotensin II receptor blockers; ASA American Society of Anesthesiologists; BMI Body Mass Index; Ca Calcium; DAP diastolic arterial pressure; IOH intraoperative hypotension; MAP mean arterial pressure; SAP systolic arterial pressure
