# Supplementary figures and images for: Preoperative patient risk factors for intraoperative hypotension: a systematic review and meta-analysis
Source: Front Cardiovasc Med. 2025 Dec 5;12:1709004. doi: 10.3389/fcvm.2025.1709004 (PMC12714980; doi:10.3389/fcvm.2025.1709004)

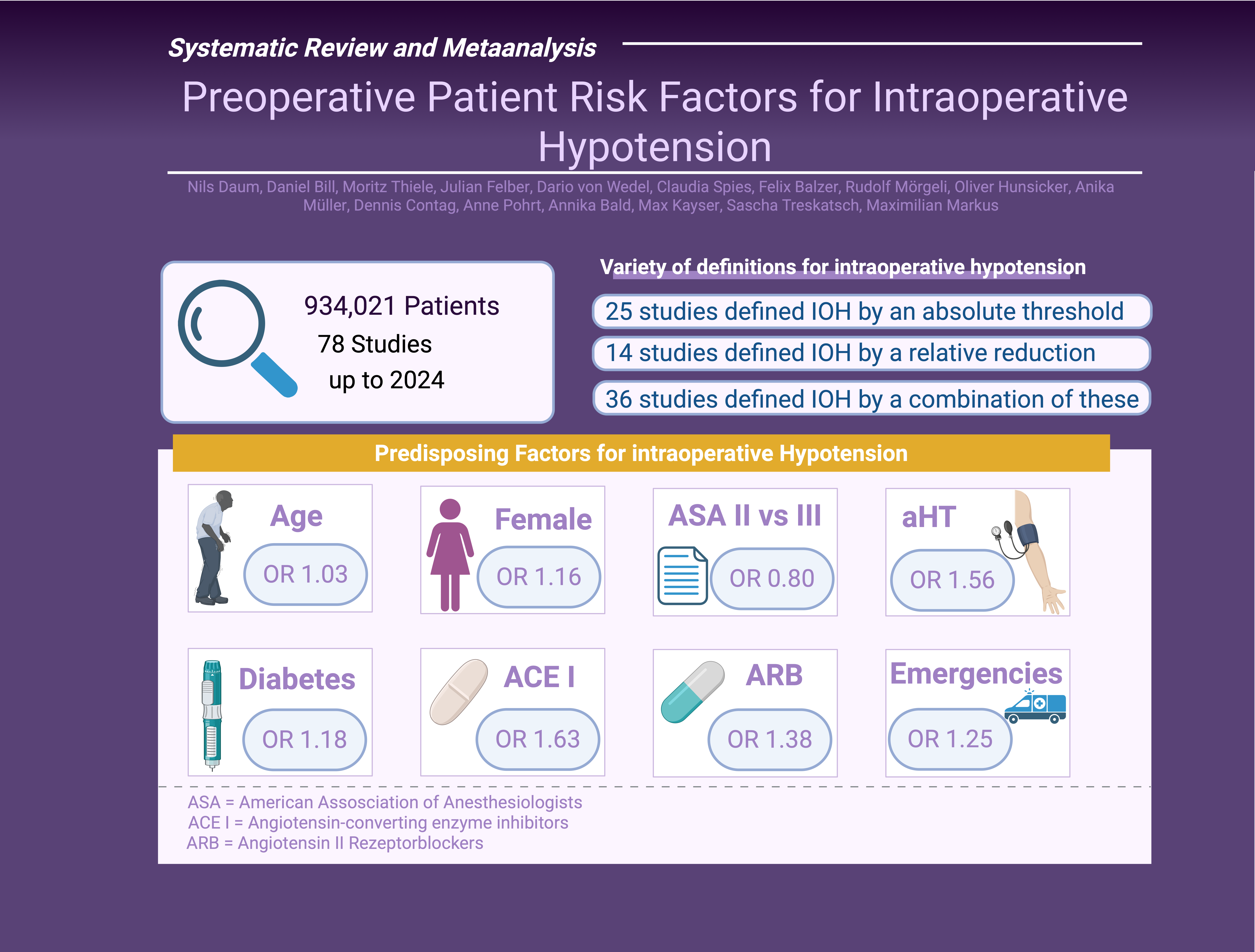

Supplement: Supplementary File 1 — Visual abstract. Created in BioRender. Markus, M. (2025), https://BioRender.com/a6gg90v. [file Supplementaryfile1.png]
